# Evaluating the Efficiency and Staff Satisfaction of the Point-of-Care IV Activated System Versus Traditional Piggyback in Administering IV Antibiotics at a Saudi Tertiary Hospital

**DOI:** 10.3390/pharmacy12050158

**Published:** 2024-10-21

**Authors:** Khaled Elshammaa, Abubakr A. Yousif, Abdullah Alshammari, Mohammed Alnuhait, Abdulmalik S. Alotaibi, Mahmoud E. Elrggal, Mohamed Hassan Elnaem

**Affiliations:** 1 Pharmaceutical Service Administration, King Abdullah Medical City, Makkah 24246, Saudi Arabia; 2 Healthcare Excellence Administration, Makkah Healthcare Cluster, Makkah 24231, Saudi Arabia; abubakrawad2020@gmail.com; 3 Pharmaceutical Practices Department, College of Pharmacy, Umm Al-Qura University, Makkah 24382, Saudi Arabia; asshammari@uqu.edu.sa (A.A.); manuhait@uqu.edu.sa (M.A.); assalotaibi@uqu.edu.sa (A.S.A.); 4 Faculty of Medicine Al-Qunfudah, Umm Al-Qura University, Makkah 24382, Saudi Arabia; elrggalme@gmail.com; 5 School of Pharmacy and Pharmaceutical Sciences, Faculty of Life and Health Sciences, Ulster University, Coleraine BT52 1SA, UK

**Keywords:** intravenous infusions, pharmacy practice, point-of-care, automated dispensing cabinets and piggyback

## Abstract

Background: This study aims to compare resource utilization and staff satisfaction between the point-of-care (POC) activated system and the traditional intravenous piggyback (PB) system in hospital pharmacy settings. Methods: Employing a pre-post quasi-experimental design from November 2019 to April 2020, the study assessed resource requirements for both the POC activated system and the traditional PB system. Additionally, a staff satisfaction survey was conducted, focusing on staff experiences related to the pharmacy preparation process and the subsequent activation of the system by nurses. Results: The POC activated system required significantly fewer full-time equivalents (FTEs) per month compared to the PB system (0.36 ± 0.05 vs. 1.56 ± 0.07; *p* < 0.0001). Using POC in automated dispensing cabinets (ADCs) reduced medication administration time and returns (6.41% vs. 1.75%; *p* < 0.0001). The staff satisfaction survey revealed greater satisfaction with the POC activated system. A subsequent analysis showed the POC activated system had a low expiration rate of 0.1% and a cost of 39 Saudi riyal, while the traditional system had higher expiration rates and cost of 46,260 SR. Conclusions: The POC activated system reduced FTEs, decreased returned medications, and enhanced staff satisfaction compared to the PB system.

## 1. Introduction

In hospital pharmacy practice, the sterile compounding of medications is a critical operation. This process involves pharmacists and pharmacy technicians who are entrusted with the responsibility of compounding and dispensing sterile products. Their role is crucial in ensuring the accuracy of ingredient identity, purity, integrity, strength, stability, and compatibility, and the sterility of these preparations [1].

The plethora of intravenous (IV) medication delivery systems presents a complex choice in hospital environments. This variety requires comparative studies to evaluate each system’s resource demands, waste generation, and user-friendliness. Common systems include piggyback (PB) containers, point-of-care (POC) activated systems, syringe pumps, and outsourced compounding services [2]. Mostly used in hospital pharmacies for sterile preparations, the PB container system requires a series of aseptic steps, including reconstitution, dilution, and admixing with the appropriate diluent, which engage a multitude of personnel and diverse equipment [3,4,5]. However, the POC activated system provides an efficient method by connecting a medication vial to a minibag. It streamlines the process of preparing IV solutions that are ready to be mixed, thus decreasing the number of stages and personnel involved [6]. This system also offers enhanced stability and flexible storage needs, allowing it to be stored in different settings, such as in refrigeration or at room temperature. It may also be placed in automated dispensing cabinets (ADCs) for quick activation before administration [7,8].

Despite these advantages, and despite recommendations from the Institute for Safe Medication Practices (ISMP) and the Food and Drug Administration (FDA) advocating for ready-to-use products to reduce workload and errors, many healthcare settings continue to employ traditional methods [1,9]. This decision often depends on cost considerations, as the POC activated system, although efficient, can incur high expenses compared to traditional methods. However, given the significant impact of sterile admixture costs on hospital budgets, an evaluation of these systems is essential [10,11,12].

This study compares the operational efficiencies, medication waste reduction, and staff satisfaction of the traditional PB system with those of the new POC activated system. The study focuses on hospital pharmacy and nursing staff, providing vital data to guide the selection of the best-suited delivery system for use in hospital settings.

## 2. Materials and Methods

### 2.1. Study Design and Procedure

This study, conducted from November 2019 to April 2020, employed a quasi-experimental design to assess the efficacy of the PB and POC activated system in the preparation of IV medications at the King Abdullah Medical City (KAMC) pharmacy, Makkah, Saudi Arabia. It encompassed all IV medications formulated within this time frame. The research involved extracting data from the Health Information System (HIS), focusing on the time needed to prepare specific antibiotic bags, including amoxicillin/clavulanic acid, ceftriaxone, cefepime, cefazolin, cefuroxime, micafungin, meropenem, tigecycline, and vancomycin. The staff included in the study were trained, according to KAMC policy, on handling sterile preparations, including both the POC activated system and PB.

### 2.2. Full-Time Equivalent (FTE) Calculation

To calculate the FTE required to prepare IV medications using different systems, we divide the total monthly hours needed for each system (51 h for a POC activated system and 219 h in the case of PB) by the number of hours worked full-time per month (140 h).

### 2.3. Tracking of Administration Events and Outcomes

The measured outcomes focused on various aspects of antibiotic bag preparation. These included the time required each month to prepare the bags, the FTE staff needed for this task, the total number of bags prepared monthly, and the percentage of bags returned relative to the total prepared. These metrics provided comprehensive information on the efficiency and effectiveness of the antibiotic preparation process using PB and POC activated systems in the KAMC pharmacy.

### 2.4. Staff Satisfaction Survey

Staff satisfaction was evaluated using an electronic survey. To ensure survey validity, the questions were developed by adapting a similar survey from the literature by Chin et al. [6]. The survey content was reviewed by pharmacy compounding experts to ensure its relevance to our study. It was designed to target pharmacy staff and nurses involved in preparing and administering IV PB and POC activated systems. The survey comprised five questions in five domains, utilizing a Likert scale ranging from 1 (strongly disagree) to 5 (strongly agree). The questions addressed various aspects of the drug delivery process, including the availability of medications for patients, ease of administration, ease of preparation, time efficiency for other tasks, and reduced need for interdepartmental communication using the POC activated system. Participants’ responses were aggregated to calculate a mean satisfaction score for each, with higher scores indicating greater satisfaction with the POC activated system.

### 2.5. Sample Size Calculation

Calculations determined that 470 bags were needed to detect differences in FTE, and 134 administration events were required to evaluate the time efficiency of drug delivery systems. The survey targeted 93 participants, based on a population of 109 end users (19 pharmacy technicians and 90 nurses), ensuring a 95% confidence interval and a margin of error of 0.05.

### 2.6. Statistical Analysis

Statistical analysis was performed using the STATA IC 16 software package. Time efficiency data were reported as mean ± SD, and categorical variables were expressed as percentages. Depending on the data distribution, Student’s *t*-test or the Mann–Whitney U test were used for group comparisons, while the Chi-square test was utilized for categorical values. A *p*-value of less than 0.05 was considered statistically significant.

## 3. Results

We evaluated the effects of switching from the traditional PB system to the POC activated system for IV medication compounding in a hospital setting. Initially, from November 2019 to January 2020, the PB system was used. Following this, in February 2020, we integrated the POC activated system to administer various antibiotics. Our analysis included workload, FTE requirements, and medication return rates, as detailed in Table 1.

The introduction of the POC activated system resulted in a 72.6% reduction in the rate of medication returns and a 66.7% decrease in monthly staff hours. Additionally, we examined 202 emergency department STAT orders to assess the efficiency of different dispensing methods, including IV PB with porters, IV PB through ADCs, and IV POC activated systems within ADCs. The findings in Table 2 revealed a substantial improvement in time efficiency when keeping the medication as a POC activated system over dispensing IV PB with a porter (*p* = 0.002). However, the efficiency between the POC activated system in ADCs and the PB system in refrigerated ADCs did not show significant differences, indicating comparable effectiveness in these two methods. These findings underscore the operational advantages of the POC activated system in reducing medication returns and enhancing the efficiency of STAT order processing.

Furthermore, we also measured the cost of items with short stability that are difficult to recycle after being returned for whatever specific reason. A subsequent analysis over 6 months, focusing on waste reduction and cost-effectiveness, examined five specific medications (Meropenem, Micafungin, Ceftazidime-Avibactam, Amoxicillin-Clavulanic Acid, and Tigecycline) and prepared using two different systems. The aim was to reveal the advantages and drawbacks associated with the POC activated system and traditional PB System. The POC activated system demonstrated a remarkably low expiration rate of 0.1%, resulting in 39 Saudi riyals (SR) associated with expired medications within three months (Table 3).

In contrast, the traditional IV PB system showed higher expiration rates for each medication studied: Tigecycline (2.1%), Micafungin (2.5%), Ceftazidime-Avibactam (4.3%), Meropenem (1.6%), and Amoxicillin-Clavulanic Acid (0.6%). These expired medications resulted in a substantial cost burden over a 3-month period, totaling 46,260 SR. The additional cost of using the POC activated system to eliminate waste due to expired medications was calculated for each of the above medications, with a total cost of 192,729 SR (Table 4).

### Staff Satisfaction

**Figure 1:**
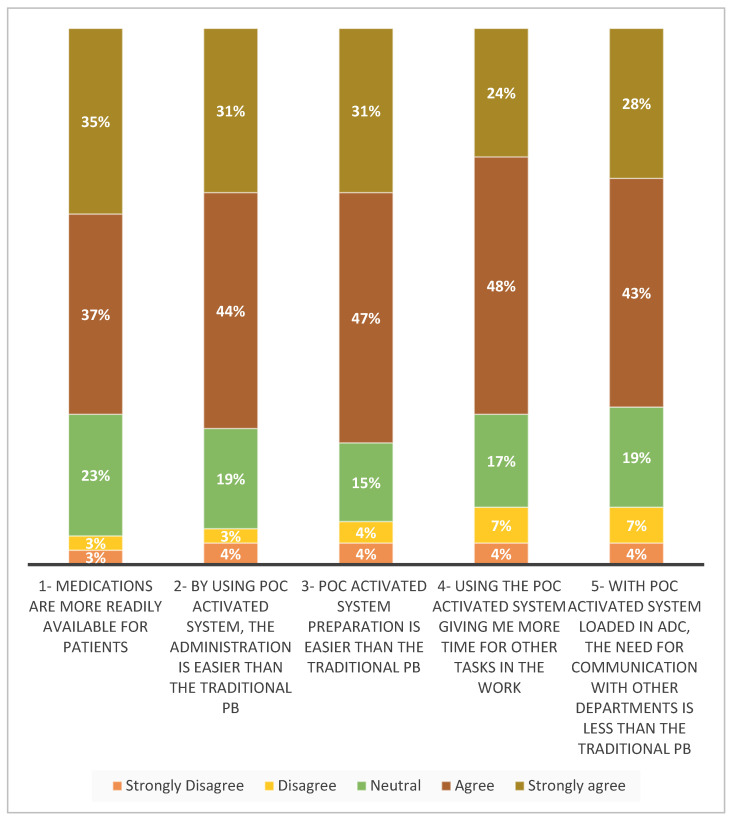
Nurses’ satisfaction with the use of POC activated system.

In our survey assessing staff satisfaction with the POC activated system, a total of 75 nursing department staff participated, consisting of 33% male and 67% female participants, with an average age of 33.03 (±4.01) years. The survey revealed that the overall satisfaction score among nurses for the POC activated system was 3.91 (±0.83) out of 5. This score indicates a higher level of satisfaction with the POC activated system compared to the traditional PB method (as shown in Figure 1).

**Figure 2:**
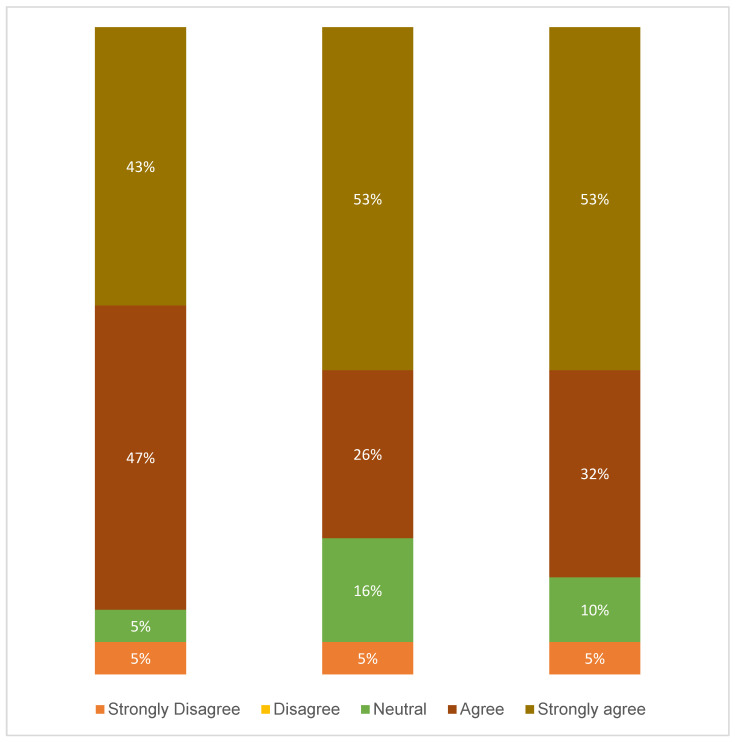
Pharmacy technicians’ satisfaction with the use of POC activated system.

Furthermore, after excluding statements 1 and 2 from the nursing department survey due to their irrelevance to the work remit of pharmacy technicians, responses from 19 pharmacy technicians were analyzed. The demographic composition of this group was 37% male and 63% female, with an average age of 35.89 (±5.85) years. Pharmacy technicians reported an overall mean satisfaction score of 4.23 (±1.04), demonstrating high satisfaction with the POC activated system (see Figure 2 for detailed insights). These findings suggest a favorable reception for the POC activated system among nursing and pharmacy staff, reflecting its operational effectiveness and user-friendliness.

## 4. Discussion

Our study, focusing on the use of the PB and POC activated systems for IV medications in a hospital setting, revealed significant operational efficiencies. In particular, the use of the POC activated system demonstrated a reduction in human resources despite a higher volume of medication preparations, aligning with other international research on resource efficiency in medication preparation [2,6,12].

The results of the study showed that the POC activated system decreased the time required for medication preparation compared to the traditional PB method, resulting in an approximate three times reduction. Furthermore, the rate of medication returns was significantly decreased for the POC activated system, roughly three times less compared to PB. These results are consistent with the findings of a prior study, which reported a 50% reduction in the time taken to administer medication after the adoption of POC activated systems [6]. Another study also reported a 30% reduction in the time nurses required for the administration of dobutamine. The findings highlight the effectiveness and decreased burden provided by the POC method [12].

The subsequent analysis focusing on waste reduction and cost-effectiveness revealed that the traditional PB system resulted in a total cost of 192,729 SR over three months due to expired medications. However, a crucial consideration is the cost of preparation with the POC activated system. For some medications (Meropenem, Ceftazidime-Avibactam, Amoxicillin-Clavulanic Acid, and Tigecycline), the cost of preparation with the POC activated system outweighed the potential savings from reduced expired medications. This highlights the importance of medication-specific analysis when evaluating cost-effectiveness.

When comparing the POC activated system to the traditional PB system, it is important to consider the acquisition costs of the medications involved. Although the POC activated system may have a higher initial setup cost, it can save money on medications that are often wasted in the PB systems. This is particularly true for expensive medications, such as Micafungin. Overall, the choice between the POC activated system and PB requires a detailed cost-effectiveness analysis that extends beyond expired cost.

What has been received from nurses about the use of the POC activated system demonstrates a considerable degree of satisfaction. Nurses have reported that the POC activated system facilitates medication preparation and delivery in a simpler and safer manner compared to the PB system. Additionally, they found the POC activated system to be more easily accessible. The response of the pharmacy staff mirrored similar thoughts. This is consistent with another study conducted with 25 nurses, which emphasized the benefits of the POC activated system. These advantages include time efficiency, less aerosol production during preparation, reduced likelihood of needle stick injuries, and lower chance of contamination due to fewer manual handling steps involved in IV medication preparation. The data indicate that personnel value the ease and time-saving benefits of the POC activated system [6,12].

Additionally, using the POC activated system with ADCs streamlined the dispensing and return processes, reducing staff time and ensuring faster availability of IV medication for patients, which can decrease labor costs and improve patient outcomes.

Finally, when any institution is willing to adopt the POC activated system, we recommend a stepwise implementation process including a cost-benefit analysis tailored to the institution’s medication needs, procurement of appropriate equipment, and staff training. We also recommend monitoring key performance indicators, such as preparation time, expired preparation percentages, and staff feedback, to ensure the efficient implementation of the system.

Although our findings are promising, the limitations must be acknowledged. This research was conducted at a single center; thus, repeating this work on larger scale with a broader multicenter approach would offer more generalizable results. The study did not measure microbiological infection rates between the POC activated system and PB. These limitations highlight areas for future research, including a pharmacoeconomic assessment of these systems and the exploration of alternative drug delivery methods such as pneumatic tubes. Such research should incorporate a comprehensive cost analysis, including both preparation and acquisition costs, to provide a better understanding of the economic impact of these systems for decision-makers in pharmaceutical services [12,13].

## 5. Conclusions

The POC activated system offers advantages in reducing waste and improving time for compounders. However, a cost-effectiveness analysis considering medication-specific factors, long-term impact, and implementation costs is crucial before adoption in a hospital pharmacy setting. Balancing efficiency, waste reduction, and cost requires the consideration of various factors to ensure optimal medication preparation practices.

## Figures and Tables

**Table 1:** Workload and FTE associated with drug delivery systems.

Drug Delivery System	Before Using the POC Activated System	After Using the POC Activated System	Statistical Comparison
Total pharmacy preparations of medications meeting the inclusion criteria within three months ^a^	15,759	18,373	Not required
Time needed for preparation per month, mean (SD), hours	219 (10.5)	51 (7)	*p* < 0.0001
FTE per month, mean (SD)	1.56 (0.07)	0.36 (0.05)	*p* < 0.0001
Preparations for IV medications stored as POC activated system in ADC of specific units within three months ^b^	8449	10,207	Not required
Total returned preparations from the IV medication group prepared as POC activated system and stored within ADCs, *n* (%)	541 (6.4)	179 (1.75)	*p* < 0.0001
The time needed to check the returned preparations per month, mean (SD), hours	4.5 (0.2)	1.5 (0.9)	*p* = 0.02

Abbreviations: FTE, full-time equivalent; POC, point of care; ADC, automated dispenser cabinet. ^a^ Amoxicillin/Clavulanic acid, Ceftriaxone, Cefepime, Cefazolin, Cefuroxime, Micafungin, Meropenem, Tigecycline, and Vancomycin. ^b^ Cefepime, Ceftriaxone, and Meropenem for critical care, emergency, hematology, oncology, and specialized surgical units.

**Table 2:** The time needed for STAT order administration.

Dispensing Method	Medication Dispensed from the Pharmacy as PB by a Porter (*n* = 67)	Medication Stored as PB in a Refrigerator Connected to an ADC (*n* = 68)	Medication Stored as a POC Activated System in an ADC (*n* = 70)
The median time needed for the administration (IQR)	51 min (35–79.8)	35.5 min (23–64.5)	33 min (18–63)

**Table 3:** Point-of-care (POC) activated system.

Medication	Preparation	Expired	Expired %	Expired Cost (SR)	Month
Tigecycline	443	0	0	0	10
Tigecycline	382	0	0	0	11
Tigecycline	799	0	0	0	12
Micafungin	298	0	0	0	10
Micafungin	326	0	0	0	11
Micafungin	455	0	0	0	12
Ceftazidime-Avibactam	288	0	0	0	10
Ceftazidime-Avibactam	305	0	0	0	11
Ceftazidime-Avibactam	238	0	0	0	12
Meropenem	2076	0	0	0	10
Meropenem	3560	3	0.1	39	11
Meropenem	4543	0	0	0	12
Amoxicillin-Clavulanic	62	0	0	0	10
Amoxicillin-Clavulanic	66	0	0	0	11
Amoxicillin-Clavulanic	52	0	0	0	12

**Table 4:** Cost of using POC activated system instead of PB.

Medication	Preparation	Expired	Expired %	Expired Cost (SR)	Month	Additional Cost if POC Activated System Was Used
Tigecycline	358	9	2.5	1179	1	6086
Tigecycline	395	4	1.0	524	2	6715
Tigecycline	344	10	2.9	1310	3	5848
Micafungin	209	2	1.0	1750	1	3553
Micafungin	384	14	3.6	12,250	2	6528
Micafungin	532	16	3.0	14,000	3	9044
Ceftazidime-Avibactam	290	17	5.9	6834	1	4930
Ceftazidime-Avibactam	270	10	3.7	4020	2	4590
Ceftazidime-Avibactam	203	7	3.4	2814	3	3451
Meropenem	2418	81	3.3	1053	1	41,106
Meropenem	3242	15	0.5	195	2	55,114
Meropenem	2544	25	1.0	325	3	43,248
Amoxicillin-Clavulanic	30	0	0.0	0	1	510
Amoxicillin-Clavulanic	64	0	0.0	0	2	1088
Amoxicillin-Clavulanic	54	1	1.9	5.5	3	918

## Data Availability

All data generated or analyzed during this study are included in this published article.

## Abbreviations

Point-of-Care (POC) activated system: A small bag (50–250 mL) of sterile IV solution with an adaptor that allows a vial of medicine with a 13 mm or 20 mm closure to be connected without being dissolved. The vial/bag system creates a physical barrier between components (fluid and medication) that can be activated to mix ingredients; Piggyback (PB): Medication dissolved in a small volume of IV solution (50–250 mL in a mini-bag); Automated Dispensing Cabinet (ADC): A decentralized medication distribution system that provides computer-controlled storage, distribution, and tracking of drugs at the POC in patient care units; STAT order: The highest priority order and life-threatening situation that need to be dealt with immediately or within a limited time frame; Full-time equivalent (FTE): A worker employed full-time; ISMP: Institute for Safe Medication Practices; FDA: Food and Drug Administration; Health Information System (HIS): System designed to manage healthcare data; IV: Intravenous.
